# Carbon Nanotubes and Chronic Granulomatous Disease

**DOI:** 10.3390/nano4020508

**Published:** 2014-06-23

**Authors:** Barbara P. Barna, Marc A. Judson, Mary Jane Thomassen

**Affiliations:** 1 Division of Pulmonary, Critical Care and Sleep Medicine, East Carolina University, Brody Medical Sciences Building, 600 Moye Blvd. Rm. 3E-149, Greenville, NC 27834, USA; E-Mail: barnab@ecu.edu; 2 Division of Pulmonary and Critical Care Medicine, Albany Medical College, MC-91, 47 New Scotland Avenue, Albany, NY 12208, USA; E-Mail: judsonm@mail.amc.edu

**Keywords:** carbon nanotubes, sarcoidosis, alveolar macrophages

## Abstract

Use of nanomaterials in manufactured consumer products is a rapidly expanding industry and potential toxicities are just beginning to be explored. Combustion-generated multiwall carbon nanotubes (MWCNT) or nanoparticles are ubiquitous in non-manufacturing environments and detectable in vapors from diesel fuel, methane, propane, and natural gas. In experimental animal models, carbon nanotubes have been shown to induce granulomas or other inflammatory changes. Evidence suggesting potential involvement of carbon nanomaterials in human granulomatous disease, has been gathered from analyses of dusts generated in the World Trade Center disaster combined with epidemiological data showing a subsequent increase in granulomatous disease of first responders. In this review we will discuss evidence for similarities in the pathophysiology of carbon nanotube-induced pulmonary disease in experimental animals with that of the human granulomatous disease, sarcoidosis.

## 1. Granulomatous Lung Disease

### 1.1. Background

Granulomatous diseases represent a world-wide health problem. Granuloma formation is a host response designed to sequester and destroy an injurious substance or infectious organism (reviewed in [1]). The granuloma itself is a complex, multicellular structure generated by components of the innate immune system (reviewed in [2]). Granulomas may form in the lung in response to environmental stimuli of a broad nature, including intracellular pathogens (mycobacteria, fungi), chemically inert materials such as silica, beryllium, or talc, and organic antigens as in hypersensitivity pneumonitis. Initial formation of the granuloma involves release of a large repertoire of pro-inflammatory mediators designed to recruit neutrophils and other cells into the area. If neutrophils cannot destroy the substance, it is phagocytized by macrophages which release additional pro-inflammatory cytokines and chemokines. This cytokine storm recruits T cells, natural killer (NK) cells, and dendritic cells so that any digested antigenic components of the substance can be presented to activate an adaptive immune response [3]. Failure of macrophages to destroy the substance within the granuloma results in a chronic inflammatory pulmonary condition that can have significant morbidity. Chronic granulomatous disease can occur in the presence or absence of a detectible adaptive immune response.

### 1.2. Sarcoidosis: Characterization

Sarcoidosis is a multi-organ, inflammatory granulomatous disease thought to occur as a result of exposure to an unidentified, poorly soluble environmental antigen(s). The antigenic substance triggers a host response which is related to genetic/epigenetic susceptibility factors [4]. As discussed below, many potential putative sarcoidosis “antigens” have been identified, but none has been clearly confirmed as a direct cause of the disease. The diagnosis of sarcoidosis is usually established by clinical history, a biopsy revealing non-caseating granulomas with adjacent inflammatory cell infiltrates, and negative studies for active infection. Currently there are no definitive biomarkers available for therapeutic targets or for assistance in the clinical evaluation of sarcoidosis patient prognosis, responses to therapy, or disease management.

### 1.3. Sarcoidosis: Epidemiology and Potential Etiology

The prevalence and incidence of sarcoidosis varies by ethnicity and sex. In the United States, the adjusted annual incidence is roughly three times higher in African-Americans than in white Americans [5,6]. Sarcoidosis is more common in females than in males by a ratio of less than two to one [7]. The prevalence of sarcoidosis also appears to vary in different geographic locations [6,8].

Several studies have reported microbial antigens (e.g., mycobacterium, propionibacterium acnes) in sarcoidosis tissue or lung, and blood cell reactivity to antigen challenge *in vitro* [9,10,11,12,13,14,15]. However, some studies have found similar carriage rates of organisms suspected to cause sarcoidosis in control subjects without disease [16]. In contrast, other studies have not confirmed the presence of potential sarcoidosis pathogens in sarcoidosis subjects [17,18].

Multiple environmental risk factors have been linked to sarcoidosis, including dose-related exposure to wood-burning stoves, fireplaces, and firefighting [4,19,20,21,22]. The atmosphere of such environmental conditions might well harbor combustion-generated contaminants composed of carbon nanotubes. Related to these findings, the incidence of “sarcoidosis-like” pulmonary granulomatous disease was reported to be increased in New York City Fire Department workers who were involved in the September 2001 World Trade Center (WTC) disaster where inhalation exposure to combustion materials was “intense” [23,24]. More recent follow-ups of some 20,000 WTC responders have detected sarcoidosis-like granulomatous disease at higher levels than expected in non-firefighting individuals as well [25]. Of interest was the observation that carbon nanotubes of various sizes and lengths were present in WTC dust samples and in lung tissues of affected exposed individuals [26]. Extensive analyses of dusts/aerosols generated by the WTC collapse resulted in four major categories of components: (1) particulate matter (calcium carbonate and silica) and fibers (asbestos, glass, gypsum); (2) organic pollutants including polycyclic aromatic hydrocarbons; (3) gases (carbon dioxide, hydrogen sulfide, diesel exhaust fumes, combustion byproducts), and (4) heavy metals [24,27]. Many of these components such as particulate matter, fibers, and combustion byproducts have been previously associated with cases of sarcoidosis-like disease [24]. Other materials known to produce granulomatous reactions such as beryllium, zirconium, or tungsten, were not detectable in WTC dusts [27]. Thus, while WTC dust exposure has been clearly linked to increased incidence of respiratory disease [28], the identity of WTC dust component(s) responsible for increased cases of sarcoidosis-like granulomatous disease remains unknown. Despite the WTC studies and other large epidemiologic and genetic studies, no definitive etiology for sarcoidosis has emerged [29,30,31].

## 2. Animal Models of Carbon Nanotube-Mediated Lung Disease

### 2.1. Effects of Carbon Nanotubes

While nanomaterials such as carbon nanotubes have great potential in fields such as drug delivery and disease imaging, standardized means to determine potential toxicities have not yet been established (reviewed in [32,33]). Carbon nanotubes represent an arrangement of C60 atoms in an elongated cylindrical structure [34]. Interestingly, the history of carbon nanotube discovery is a complex tale which may go back well over a century. As colorfully described by Monthioux and Kuznetsov [35] the first mention of such structures may have appeared in Russia, in a 1952 volume of the Journal of Physical Chemistry. Not until 1993, however, were preparations of single wall carbon nanotubes (SWCNT) first detailed by two different investigative groups [35]. SWCNT can be massed into multi-wall carbon nanotubes (MWCNT) [34]. Outside of manufacturing, combustion-generated MWCNT and other carbon nanoparticles are ubiquitous within the environment, and have been detected in vapors from diesel fuel, methane, propane, and natural gas (reviewed in [36]). Thus, the importance and impact of carbon particles in our environment might be underestimated as a causative agent and/or factor involved in the development of respiratory illnesses.

Granulomatous inflammation and fibrosis have been reported in rodent models as a response to intratracheal administration of SWCNT or MWCNT [37,38,39]. Several factors have been cited as contributors to these findings. Pulmonary granulomatous changes have been associated with large agglomerates or aggregates of carbon nanotubes [40,41,42]. A comparative study of lung tissues in inhalation versus instillation reported smaller particle size and diminished pulmonary inflammation with inhalation [43]. The authors suggested that differences might be due to the reduced size and aggregation of inhaled carbon nanotubes. However, investigations carried out to determine long-term effects of inhalation versus instillation have demonstrated similar results with both techniques [44,45]. In animals receiving dispersed carbon nanotubes, granulomatous structures have not been observed, although increased collagen disposition was detected indicative of a fibrotic response [41,46].

Carbon nanotubes have also been implicated in triggering inflammasome formation [47,48,49]. Inflammasomes are part of innate immunity and consist of large intracellular protein complexes that regulate proteolytic activation of the proinflammatory cytokines, IL-1β and IL-18 [50,51]. These structures can be activated by microbial triggers as well as non-microbial materials such as asbestos and silica [52]. Inflammasome activation is now recognized as a contributor to a number of chronic lung diseases such as idiopathic pulmonary fibrosis, chronic obstructive lung disease, and asthma [51].

The physical and chemical properties of carbon nanotubes strongly influence pulmonary toxicity. Metal contaminants in commercial carbon nanotube preparations have been implicated in nanotube toxicity [53]. Hamilton *et al.* [47] presented both *in vitro* and *in vivo* results suggesting that inflammatory responses increased with MWCNT diameter and length. This group also found that nickel contamination of nanotubes activated inflammasomes, exacerbated lung pathology and promoted release of pro-inflammatory cytokines from alveolar macrophages [47]. Other properties of carbon nanotubes associated with inflammasome formation and toxicity included double-walled [49] and long-needle-like structures [48]. In contrast, modification of carbon nanotubes with polymer coatings [54] or other biocompatible materials has been reported to reduce pulmonary toxicity (summarized in [55,56]).

Currently, there is ample evidence that carbon nanotubes provoke pulmonary innate immune and inflammatory responses [37,38,39,48,49], but not adaptive immune responses (reviewed in [57]). Results of MWCNT interaction with existing immune system adaptive responses, however, are controversial. Inhalation of MWCNT (0.3–5 mg/m^3^) did not cause significant pulmonary changes but did increase TGFβ levels in bronchoalveolar lavage fluid which when applied *in vitro* to splenocytes from untreated mice, suppressed T cell activity [58]. In another inhalation model, Staal *et al.* [59] observed decreased allergen-specific IgE responses in sensitized animals. In contrast, Ronzani *et al.* [60], reported that instilled MWCNT elevated Th2 responses in a house dust mite allergy model. Finally, Ryman-Rasmussen *et al.* [61] found no significant pulmonary changes after MWCNT inhalation (100 mg/m^3^) except in ovalbumin-sensitized animals challenged with antigen. MWCNT treatment then increased pulmonary fibrosis and levels of the fibroblast growth factor, PDGF-AA, but had no effects on TGFβ or IL-13. These studies suggest that complications in both innate and adaptive immune functions may occur after exposure to carbon nanotubes.

### 2.2. The MWCNT-Induced Chronic Granuloma Model

Our major goal has been to improve understanding of sarcoidosis pathophysiology. The relatively recent reports of carbon nanotubes in environments favoring sarcoidosis incidence has been the impetus for us to develop a model of MWCNT-mediated lung disease that might have applicability to sarcoidosis. To our knowledge, no other MWCNT models have been used to study sarcoidosis. This model has proven to be extremely interesting with many pathologic changes identical to those found in sarcoidosis lung. Because most granulomatous diseases are chronic, and inciting factors may be unknown, there are no ideal animal models with which to investigate mechanisms of persistent granulomatous inflammation. Our first report described a model in which instilled MWCNT led to granulomatous lung inflammation persisting up to 90 days [62]. The long-term chronicity of this carbon nanotube granuloma model is relevant to the exploration of mechanisms in human chronic granulomatous disease. Previously, major findings related to granulomatous inflammation were derived from studies using an acute murine granuloma model in which antigen-bound sepharose beads were injected intravenously into pre-sensitized animals [63,64]. However, such models are limited in that granulomatous inflammation forms and resolves within 14–20 days. Also of consideration was the delivery route, a tail vein injection of antigen bound-sepharose beads that lodged in the pulmonary circulation rather than directly in the alveolar space.

Dosage of reagent is problematic in all animal disease models particularly in the case of sarcoidosis where the inciting antigen(s), mode of induction, and minimum dose exposure have not been established for the human disease. Moreover, although murine models are ubiquitously studied, many basic biological differences exist in both the innate and adaptive immune responses of mice and man [65]. Further, pulmonary disease models must also take into account differences in murine and human bronchoalveolar lavage fluid proteins [66]. Despite these problems, murine models of pulmonary disease such as the sepharose bead granuloma model mentioned above, have provided valuable data on inflammatory granuloma formation [63,67].

The MWCNT preparation used in our model exhibited a dominant range of nanotube diameters from 20 to 40 nm and a net metal catalyst (Fe) content of less than 1.0% [62]. MWCNT (25–100 μg) were administered as a bolus in a 35% surfactant-saline solution via oropharyngeal instillation. The dosage we selected was within the range inducing granulomas in a study by Lam *et al.* [68] using intratracheally administered SWCNT. Inhalation studies have demonstrated that exposure of rats to aerosols containing 0.1 mg/m^3^ MWCNT can yield pulmonary granulomatous inflammation [69]. When compared to an aerosol delivery method, however, retropharyngeal instillation may be superior as it achieves full and consistent delivery, with decreased variability between exposures [43,67]. In our model, small granulomatous reactions surrounding MWCNT aggregates were detectable by 10 days post instillation with larger reactions persisting for up to 90 days. Bronchoalveolar lavage (BAL) of mice revealed MWCNT within alveolar macrophages [62].

The major goal in the initial studies of our chronic MWCNT model was to document pathways involved in granuloma formation by analyzing potential mediators in granuloma/non-granuloma lung tissues and BAL cells. Granulomatous foci and non-granulomatous tissues were collected by laser capture microdissection (LCM) for mRNA quantitation. Protein expression in lung tissues or BAL cells was evaluated by immunohistochemistry. Results showed elevated expression of osteopontin, a cytokine reported to be critical for granuloma formation [70], in granulomatous tissues and in BAL cells. Other granuloma-promoting factors found to be elevated were cell adhesion molecules (integrins) which are necessary for the fusion of macrophages into multinucleated giant cells within the granuloma structure [71]. Similarly, matrix metalloproteinases (MMPs) such as MMP-12 were also elevated in granulomatous tissue and in BAL cells. MMP-12 is a contributor to lung remodeling and targets elastin [72]. Overexpression has been reported to increase granuloma size in experimental animals [73]. Elevated MMP12 was also noted in a SWCNT model showing granulomas [74]. In our model, we also observed a concomitant decrease in Tissue Inhibitor of MMPs (TIMP)-3, an inhibitor of MMP-12 [75], suggesting that MMP-12 activity was unopposed by tissue inhibitors. These findings documented the formation of well-organized granulomas with time-related increases in granuloma size and mediator expression.

### 2.3. Parallel Findings in the MWCNT Model and Sarcoidosis

A review of selective findings from the chronic MWCNT granuloma model reveals many pathophysiologic similarities to human sarcoidosis (Table 1). With respect to granuloma formation, osteopontin and MMP-12 elevations noted in the MWCNT model have also been reported as characteristics of sarcoidosis granulomatous tissues [76,77].

**Table 1:** Comparative findings in the multiwall carbon nanotube (MWCNT) chronic granuloma model and human sarcoidosis.

Mediators/Regulators of Inflammation or Damage	Pulmonary Findings	
	MWCNT Model	Sarcoidosis
Osteopontin	Granuloma tissue; BAL * cells and fluids, [62]	Granuloma tissue [76]
MMP-12	Granuloma tissue, BAL * cells [62]. Lung tissue [74]	Granuloma tissue [77]
CCL2 (MCP-1)	Granuloma tissue [62]. Lung tissue [74]	BAL * fluids [78,79]
CCL5 (Rantes)	BAL * cells (unpublished data)	BAL * fluids [78,79]
TNFα	Granuloma tissue [62]	Trans-bronchial biopsy tissues [80]
IFN-γ	Granuloma tissue; BAL * cells [81]	Granuloma tissue [82]; BAL * cells [83]
PPARγ	Alveolar macrophages [81]	Alveolar macrophages [84]
Twist1	Alveolar macrophages [85]	Alveolar macrophages [85]

Note: * Bronchoalveolar Lavage.

A survey of pro-inflammatory cytokines and chemokines in the MWCNT model also demonstrated close similarities with sarcoidosis lung (Table 1). Elevated TNFα and the chemokine CCL2 (MCP-1) were noted in murine granulomatous tissue at 60 and 90 days post MWCNT instillation [62]. More recent studies in progress have also detected CCL5 (Rantes) production at 60 days after MWCNT instillation (unpublished data). All three of these pro-inflammatory mediators are known to be elevated in sarcoidosis granulomatous tissue and BAL cells [78,80] and are indicators of NF-κB activation which we have documented previously in sarcoidosis [84].

In our next study of the MWCNT model, we examined the role of the transcription factor, peroxisome proliferator-activated receptor gamma (PPARγ). While this transcription factor is a critical regulator of lipid and glucose metabolism, PPARγ also exerts negative effects on many pro-inflammatory genes [86]. Our previous studies had established that PPARγ binding activity and expression were deficient in alveolar macrophages from sarcoidosis patients [84]. Our first aim in the MWCNT model was to determine whether MWCNT instillation would also cause a reduction in alveolar macrophage PPARγ from wild-type mice. All studies were terminated at 60 days after MWCNT instillation. Results from wild-type mice proved to be virtually identical to those we observed in sarcoidosis, with alveolar macrophages showing significantly depressed PPARγ activity and expression [81]. Our second aim in the study was to determine whether pre-existing PPARγ deficiency would affect production of granuloma structures and inflammatory mediators. To this end, we utilized macrophage-specific PPARγ KO conditional mice. Results from MWCNT-instilled PPARγ KO mice demonstrated a more extensive granuloma formation and higher levels of osteopontin and pro-inflammatory cytokines (IFN-γ, CCL2) in granulomatous tissues and BAL cells than in similarly treated wild-type mice [81]. Thus results suggested that PPARγ functions as a negative regulator of chronic granulomatous inflammation in the lung.

In our latest publication, we utilized the MWCNT model to explore expression of the Twist1 transcription factor in alveolar macrophages. Initially, we had carried out gene array analysis of BAL cells in sarcoidosis patients and healthy human controls to ascertain patterns of macrophage activation phenotypes M1 and M2 [85]. Findings showed a predominantly M1 phenotype in sarcoidosis alveolar macrophages together with a significant increase in Twist1 gene expression. Twist1 has been shown to be a regulator of embryonic mesenchymal development [87] but its possible function in alveolar macrophages has not been ascertained. Gene array data were confirmed by quantitative mRNA analysis of sarcoidosis BAL cells and protein expression was found to be localized to alveolar macrophages. To determine whether Twist1 expression could be a general marker of inflammatory alveolar macrophages, wild-type and macrophage-specific PPARγ KO mice received instillations of MWCNT and were analyzed 60 days later. Results were similar to findings in sarcoidosis: Twist1 protein was elevated in both wild-type and PPARγ KO alveolar macrophages after MWCNT instillation. Levels of Twist1 expression overall were higher in PPARγ KO mice than wild-type, an observation that coincided with previous findings showing higher levels of inflammatory mediators in PPARγ KO versus wild-type mice [81]. While functional studies of Twist1 are still in progress, the current data confirm that Twist1 may coincide with an inflammatory M1 alveolar macrophage phenotype in both mouse and man.

## 3. Summary and Conclusions

Human granulomatous diseases are chronic in nature and comprise a world-wide health problem. In many cases such as sarcoidosis, the etiology may be unknown. Efforts to study mechanisms of granulomatous disease have been hampered by the lack of a chronic pulmonary disease model. In studies of carbon nanotube toxicity, investigators have frequently reported persistent granulomatous structures within the lungs of experimental animals after instillation of carbon nanotubes. Based upon such studies, we developed a MWCNT model of chronic granulomatous lung disease which has proved to exhibit numerous pathophysiologic characteristics similar to those of human sarcoidosis. The possibility of carbon nanotube contribution to human granulomatous diseases such as sarcoidosis is an important environmental issue but has not been addressed previously. Overall, data from the chronic MWCNT model suggest that this model has a broad range of applicability for probing mechanisms of inflammation and granuloma persistence, as well as the potential for exploring novel therapeutic approaches to chronic granulomatous disease.
